# Supplementary material for: Risk of acute myocardial infarction among new users of chondroitin sulfate: A nested case-control study
Source: PLoS One. 2021 Jul 12;16(7):e0253932. doi: 10.1371/journal.pone.0253932 (PMC8274913; doi:10.1371/journal.pone.0253932)
Supplement: S1 File — (DOCX) [file pone.0253932.s001.docx]

**SUPPLEMENTARY MATERIAL**

**RISK OF ACUTE MYOCARDIAL INFARCTION AMONG NEW USERS OF CHONDROITIN SULFATE: A NESTED CASE-CONTROL STUDY**

Ramón Mazzucchelli^1^, Sara Rodríguez-Martín ^2,3^, Alberto García-Vadillo^4^, Miguel Gil ^5^, Antonio Rodríguez-Miguel^2,3^, Diana Barreira-Hernández^2,3^, Alberto García-Lledó ^6,7^ and Francisco J. de Abajo ^2,3^

1. Rheumatology Unit, Hospital Universitario Fundación Alcorcón, Alcorcón, 28922 Madrid, Spain; [rmazzucchelli@fhalcorcon.es](mailto:rmazzucchelli@fhalcorcon.es) (R.M);
2. Clinical Pharmacology Unit, University Hospital Príncipe de Asturias, Alcalá de Henares, 28805 Madrid, Spain; sara.rodriguezm@uah.es (S.R.M.), antonio.hupa@gmail.com (A.R.M.), dbarreirahdez@gmail.com (D.B.), [francisco.abajo@uah.es](mailto:francisco.abajo@uah.es) (F.dA.)
3. Department of Biomedical Sciences (Pharmacology), School of Medicine and Health Sciences, University of Alcalá (IRYCIS), Alcalá de Henares 28805, Madrid, Spain.
4. Rheumatology Department, Hospital Universitario La Princesa, Madrid , 28006 Madrid, Spain; garciavadilloalberto@gmail.com (A.G.V);
5. Division of Pharmacoepidemiology and Pharmacovigilance, Spanish Agency of Medicines and Medical Devices (AEMPS), 28022 Madrid, Spain; mgilg@aemps.es (M.G.);
6. Department of Cardiology, University Hospital Príncipe de Asturias, Alcalá de Henares, 28805 Madrid, Spain; josealberto.garcia@salud.madrid.org
7. Department of Medicine, University of Alcalá, Alcalá de Henares, 28805 Madrid, Spain

S1 APPENDIX. Supplementary methods

Multiple Imputation by Chained Equations (MICE) models.

MICE is a statistical method that allows the assignment of a value to variables with missing information. The imputed values are sampled from their predictive distribution based on the observed data. Patterns of missing values are classified into three categories; “missing completely at random (MCAR)”, “missing at random (MAR)” and “missing not at random (MNAR)”. Often, imputing values with a MNAR pattern can give misleading results so before deciding to perform a MICE model, researchers have to carefully study the patterns of missingness in order to rule out such MNAR. In this study, we explored the characteristics of the missing values and their relations with the observed data, and finally we accepted a MAR pattern.

Among the study population (N=140,990) there were missing values for smoking (50.0%) and Body Max Index (BMI- 39.3%). Missing values of smoking and BMI were related inversely with other cardiovascular risk factors as history of heart failure or diabetes, which may be explained by the greater tendency of the primary care physician to record exposure to tobacco or BMI in patients with comorbidities linked to these risk factors. To address missing values, we run a MICE model n-times for each variable to impute with k-cycles of stabilization every time. In this case, we set the model to run 20 times, creating 20 imputed databases, and 10 cycles to stabilize the variance before creating one imputed database. The MICE model successfully imputed all the missing values. In order to test the performance of the model, two sensitivity analyses were carried out; first, we plotted the distribution and density of all values (observed + imputed + complete), observing that the imputed values followed the original distribution of the observed and complete data in each variable; and second, we performed a “complete cases” analysis and the main estimators barely changed when compared with results from the imputed dataset and the observed dataset, supporting our initial assumption of a MAR pattern.

**S1 Table:** Risk of AMI associated with the current use of Chondroitin Sulfate (CS), using Glucosamine as the reference.

|  | Cases (%)  N=23585 | Controls (%)  N=117405 | Non-adjusted OR^*^  (95% CI) | Adjusted OR^†^  (95% CI) |
| --- | --- | --- | --- | --- |
| Non users | 22606 (95.85) | 112202 (95.57) | 1.14 (0.88-1.46) | 1.11 (0.85-1.43) |
| Current users  CS  Glucosamine  CS + Glucosamine | 82 (0.35)  72 (0.31)  7 (0.03) | 681 (0.58)  406 (0.35)  76 (0.06) | 0.67 (0.47-0.94)  1 (Ref.)  0.52 (0.23-1.17) | 0.64 (0.45-0.91)  1 (Ref.)  0.54 (0.24-1.24) |
| Recent or Past users | 818 (3.47) | 4040 (3.44) | 1.14 (0.88-1.48) | 1.05 (0.80-1.37) |

Abbreviations: CI: Confident Interval; OR: Odds ratio.

^*^Adjusted only for matching factors (age, sex, and calendar year).

^†^ Adjusted for: cerebrovascular disease (ischemic, hemorrhagic or unspecified stroke and transient ischemic attack), heart failure, angina pectoris (recorded as such, and/or use of nitrates), peripheral artery disease (PAD), hypertension, diabetes (recorded as such, and/or use of glucose-lowering medications), dyslipidemia (recorded as such, and/or use of lipid-lowering medications), rheumatoid arthritis, chronic kidney disease, asymptomatic hyperuricemia and gout, number of visits to the PCP in the year prior to the index date (as an indicator of comorbidities), body mass index (BMI), smoking, and current use of the following drugs: antiplatelet drugs, oral anticoagulants, nonsteroidal anti-inflammatory drugs (NSAIDs), paracetamol, metamizole, calcium with or without vitamin D supplements, corticosteroids, proton pump inhibitors (PPIs), H2-receptor antagonists, corticosteroids, angiotensin-converting enzyme inhibitors (ACEI), angiotensin II receptor blockers (ARB), calcium channel blockers (CCB), beta-blockers, alpha-blockers, and diuretics.

**S2 Table.** AMI risk associated with the use of SYSADOAs (Chondroitin Sulfate and Glucosamine) by sex, age, NSAID use and background cardiovascular risk.

| <70 years | | | | |
| --- | --- | --- | --- | --- |
|  | **Cases (%)**  **N=13031** | **Controls (%)**  **N=64725** | **Non-adjusted**^†^ **OR (95%CI)** | **Adjusted OR**^§^ **(95%CI)** |
| SYSADOA (all)  Non users  Current  Recent  Past | 12541 (96.24)  90 (0.69)  139 (1.07)  261 (2.00) | 62055 (95.87)  612 (0.95)  725 (1.12)  1333 (2.06) | 1 (Ref.)  0.72 (0.57-0.89)  0.93 (0.78-1.12)  0.97 (0.85-1.11) | 1 (Ref.)  0.70 (0.55-0.88)  0.88 (0.72-1.06)  0.88 (0.76-1.02) |
| Glucosamine  Non users  Current  Recent  Past | 12777 (98.05)  43 (0.33)  70 (0.54)  141 (1.08) | 63424 (97.99)  246 (0.38)  304 (0.47)  751 (1.16) | 1 (Ref.)  0.87 (0.63-1.20)  1.13 (0.87-1.47)  0.93 (0.78-1.12) | 1 (Ref.)  0.87 (0.62-1.22)  1.05 (0.80-1.38)  0.85 (0.70-1.04) |
| Chondroitin sulfate  Non users  Current  Recent  Past | 12725 (97.61)  54 (0.41)  83 (0.64)  169 (1.30) | 62960 (97.27)  420 (0.65)  500 (0.77)  845 (1.31) | 1 (Ref.)  0.62 (0.47-0.83)  0.81 (0.64-1.02)  0.99 (0.84-1.17) | 1 (Ref.)  0.61 (0.45-0.82)  0.78 (0.61-0.99)  0.90 (0.76-1.08) |
|  | | | | |
| 70+ years | | | | |
|  | **Cases (%)**  **N=10544** | **Controls (%)**  **N=52680** | **Non-adjusted**^†^ **OR (95% CI)** | **Adjusted OR**^§^ **(95% CI)** |
| SYSADOA (all)  Non users  Current  Recent  Past | 10065 (95.37)  71 (0.67)  136 (1.29)  282 (2.67) | 50147 (95.19)  549 (1.04)  590 (1.12)  1394 (2.65) | 1 (Ref.)  0.64 (0.50-0.82)  1.14 (0.95-1.38)  1.01 (0.88-1.15) | 1 (Ref.)  0.69 (0.53-0.89)  1.15 (0.95-1.41)  0.97 (0.84-1.11) |
| Glucosamine  Non users  Current  Recent  Past | 10258 (97.20)  36 (0.34)  59 (0.56)  201 (1.90) | 51160 (97.11)  236 (0.45)  312 (0.59)  972 (1.85) | 1 (Ref.)  0.77 (0.54-1.09)  0.95 (0.71-1.25)  1.05 (0.90-1.22) | 1 (Ref.)  0.85 (0.59-1.22)  0.94 (0.70-1.25)  1.02 (0.86-1.20) |
| Chondroitin sulfate  Non users  Current  Recent  Past | 10302 (97.64)  35 (0.33)  89 (0.84)  128 (1.21) | 51363 (97.50)  337 (0.64)  317 (0.60)  663 (1.26) | 1 (Ref.)  0.51 (0.36-0.72)  1.37 (1.08-1.75)  0.95 (0.78-1.15) | 1 (Ref.)  0.54 (0.38-0.77)  1.42 (1.11-1.82)  0.90 (0.74-1.10) |
|  | | | | |
| Females | | | | |
|  | **Cases (%)N=6663** | **Controls (%)N=33080** | **Non-adjusted**^†^ **OR (95% CI)** | **Adjusted OR**^§^ **(95% CI)** |
| SYSADOA (all)  Non users  Current  Recent  Past | 6232 (93.53)  71 (1.07)  115 (1.73)  245 (3.68) | 30812 (93.14)  509 (1.54)  551 (1.67)  1208 (3.65) | 1 (Ref.)  0.67 (0.52-0.86)  1.02 (0.83-1.25)  1.01 (0.87-1.16) | 1 (Ref.)  0.71 (0.54-0.92)  1.02 (0.82-1.26)  0.94 (0.81-1.10) |
| Glucosamine  Non users  Current  Recent  Past | 6409 (96.19)  32 (0.48)  53 (0.80)  169 (2.54) | 31751 (95.98)  220 (0.67)  278 (0.84)  831 (2.51) | 1 (Ref.)  0.71 (0.49-1.02)  0.93 (0.69-1.25)  1.01 (0.85-1.20) | 1 (Ref.)  0.79 (0.54-1.16)  0.91 (0.67-1.25)  0.98 (0.82-1.17) |
| Chondroitin sulfate  Non users  Current  Recent  Past | 6423 (96.40)  42 (0.63)  73 (1.10)  125 (1.88) | 31826 (96.21)  316 (0.96)  321 (0.97)  617 (1.87) | 1 (Ref.)  0.64 (0.46-0.89)  1.12 (0.86-1.44)  1.02 (0.84-1.25) | 1 (Ref.)  0.65 (0.46-0.91)  1.14 (0.87-1.50)  0.94 (0.76-1.15) |
|  | | | | |
| Males | | | | |
|  | **Cases (%)**  **N=16922** | **Controls (%)**  **N=84325** | **Non-adjusted**^†^ **OR (95% CI)** | **Adjusted OR**^§^ **(95% CI)** |
| SYSADOA (all)  Non users  Current  Recent  Past | 16374 (96.76)  90 (0.53)  160 (0.95)  298 (1.76) | 81390 (96.52)  652 (0.77)  764 (0.91)  1519 (1.80) | 1 (Ref.)  0.68 (0.55-0.85)  1.04 (0.88-1.24)  0.97 (0.86-1.11) | 1 (Ref.)  0.67 (0.54-0.85)  0.99 (0.83-1.19)  0.90 (0.79-1.02) |
| Glucosamine  Non users  Current  Recent  Past | 16626 (98.25)  47 (0.28)  76 (0.45)  173 (1.02) | 82833 (98.23)  262 (0.31)  338 (0.40)  892 (1.06) | 1 (Ref.)  0.91 (0.66-1.24)  1.13 (0.88-1.45)  0.97 (0.82-1.14) | 1 (Ref.)  0.91 (0.66-1.25)  1.06 (0.82-1.38)  0.89 (0.75-1.05) |
| Chondroitin sulfate  Non users  Current  Recent  Past | 16604 (98.12)  47 (0.28)  99 (0.59)  172 (1.02) | 82497 (97.83)  441 (0.52)  496 (0.59)  891 (1.06) | 1 (Ref.)  0.52 (0.39-0.71)  0.99 (0.79-1.23)  0.95 (0.81-1.12) | 1 (Ref.)  0.52 (0.38-0.70)  0.96 (0.77-1.20)  0.89 (0.75-1.05) |
|  | | | | |
| CV risk Low | | | | |
|  | **Cases (%)**  **N=3877** | **Controls (%)**  **N=33736** | **Non-adjusted**^†^ **OR (95% CI)** | **Adjusted OR**^§^ **(95% CI)** |
| SYSADOA (all)  Non users  Current  Recent  Past | 3798 (97.96)  14 (0.36)  32 (0.83)  33 (0.85) | 32784 (97.18)  232 (0.69)  277 (0.82)  443 (1.31) | 1 (Ref.)  0.74 (0.38-1.44)  1.31 (0.80-2.17)  0.74 (0.45-1.21) | 1 (Ref.)  0.73 (0.37-1.44)  1.40 (0.83-2.36)  0.74 (0.44-1.24) |
| Glucosamine | < 5 exposed* |  | ~~-~~ | ~~-~~ |
| Chondroitin sulfate  Non users  Current  Recent  Past | 3823 (98.61)  11 (0.28)  20 (0.52)  23 (0.59) | 33142 (98.24)  145 (0.43)  180 (0.53)  269 (0.80) | 1 (Ref.)  1.18 (0.52-2.65)  1.08 (0.59-1.98)  1.04 (0.57-1.90) | 1 (Ref.)  1.11 (0.48-2.56)  1.13 (0.61-2.11)  1.10 (0.59-2.05) |
|  | | | | |
| CV Risk Intermediate | | | | |
|  | **Cases (%)**  **N=10513** | **Controls (%)**  **N=55470** | **Non-adjusted**^†^ **OR (95% CI)** | **Adjusted OR**^§^ **(95% CI)** |
| SYSADOA (all)  Non users  Current  Recent  Past | 9982 (94.95)  96 (0.91)  148 (1.41)  287 (2.73) | 52568 (94.77)  650 (1.17)  717 (1.29)  1535 (2.77) | 1 (Ref.)  0.85 (0.66-1.08)  1.05 (0.86-1.29)  1.03 (0.89-1.19) | 1 (Ref.)  0.79 (0.61-1.02)  1.05 (0.85-1.30)  0.97 (0.83-1.13) |
| Glucosamine  Non users  Current  Recent  Past | 10211 (97.13)  47 (0.45)  69 (0.66)  186 (1.77) | 53880 (97.13)  270 (0.49)  330 (0.59)  990 (1.78) | 1 (Ref.)  1.02 (0.72-1.45)  1.04 (0.78-1.39)  1.10 (0.92-1.32) | 1 (Ref.)  1.00 (0.69-1.43)  1.07 (0.79-1.44)  1.02 (0.84-1.23) |
| Chondroitin sulfate  Non users  Current  Recent  Past | 10213 (97.15)  54 (0.51)  94 (0.89)  152 (1.45) | 53759 (96.92)  427 (0.77)  445 (0.80)  839 (1.51) | 1 (Ref.)  0.72 (0.52-0.99)  1.11 (0.86-1.44)  0.92 (0.75-1.11) | 1 (Ref.)  0.65 (0.46-0.91)  1.11 (0.85-1.45)  0.89 (0.73-1.09) |
|  | | | | |
| CV Risk High | | | | |
|  | **Cases (%)**  **N=9195** | **Controls (%)**  **N=28199** | **Non-adjusted**^†^ **OR (95% CI)** | **Adjusted OR**^§^  **(95% CI)** |
| SYSADOA (all)  Non users  Current  Recent  Past | 8826 (95.99)  51 (0.55)  95 (1.03)  223 (2.43) | 26850 (95.22)  279 (0.99)  321 (1.14)  749 (2.66) | 1 (Ref.)  0.53 (0.36-0.77)  1.00 (0.75-1.34)  0.93 (0.76-1.14) | 1 (Ref.)  0.61 (0.41-0.91)  1.04 (0.77-1.41)  0.90 (0.72-1.11) |
| Glucosamine  Non users  Current  Recent  Past | 8979 (97.65)  29 (0.32)  47 (0.51)  140 (1.52) | 27453 (97.35)  108 (0.38)  156 (0.55)  482 (1.71) | 1 (Ref.)  0.76 (0.44-1.29)  1.07 (0.71-1.61)  0.92 (0.72-1.18) | 1 (Ref.)  0.85 (0.49-1.47)  1.13 (0.74-1.73)  0.91 (0.70-1.18) |
| Chondroitin sulfate  Non users  Current  Recent  Past | 8991 (97.78)  24 (0.26)  58 (0.63)  122 (1.33) | 27422 (97.24)  185 (0.66)  192 (0.68)  400 (1.42) | 1 (Ref.)  0.40 (0.24-0.69)  0.94 (0.65-1.35)  0.88 (0.67-1.16) | 1 (Ref.)  0.48 (0.27-0.83)  1.00 (0.68-1.47)  0.85 (0.64-1.13) |
|  | | | | |
| NSAIDs current users* | | | | |
|  | **Cases (%)**  **N=2327** | **Controls (%)**  **N=10454** | **Non-adjusted**^†^ **OR (95% CI)** | **Adjusted OR**^§^ **(95% CI)** |
| SYSADOA (all)  Non users  Current  Recent  Past | 2108 (90.59)  54 (2.32)  58 (2.49)  107 (4.60) | 9497 (90.85)  311 (2.97)  247 (2.36)  399 (3.82) | 1 (Ref.)  0.78 (0.58-1.05)  1.05 (0.79-1.40)  1.22 (0.98-1.51) | 1 (Ref.)  0.78 (0.57-1.05)  0.99 (0.73-1.34)  1.11 (0.88-1.39) |
| Glucosamine  Non users  Current  Recent  Past | 2190 (94.11)  30 (1.29)  30 (1.29)  77 (3.31) | 9907 (94.77)  141 (1.35)  126 (1.21)  280 (2.68) | 1 (Ref.)  0.97 (0.65-1.45)  1.07 (0.72-1.61)  1.26 (0.98-1.63) | 1 (Ref.)  1.04 (0.69-1.57)  1.08 (0.72-1.64)  1.20 (0.91-1.57) |
| Chondroitin sulfate  Non users  Current  Recent  Past | 2214 (95.14)  24 (1.03)  35 (1.50)  54 (2.32) | 9893 (94.63)  195 (1.87)  147 (1.41)  219 (2.09) | 1 (Ref.)  0.54 (0.35-0.83)  1.05 (0.72-1.52)  1.09 (0.81-1.48) | 1 (Ref.)  0.52 (0.33-0.80)  0.97 (0.66-1.42)  0.97 (0.71-1.33) |
|  | | | | |
| NSAIDs non users (also including past users)* | | | | |
|  | **Cases (%)**  **N=15660** | **Controls (%)**  **N=80884** | **Non-adjusted**^†^ **OR (95% CI)** | **Adjusted OR**^§^ **(95% CI)** |
| SYSADOA (all)  Non users  Current  Recent  Past | 15259 (97.44)  51 (0.33)  93 (0.59)  257 (1.64) | 78565 (97.13)  406 (0.50)  496 (0.61)  1417 (1.75) | 1 (Ref.)  0.65 (0.48-0.86)  0.96 (0.77-1.20)  0.93 (0.81-1.07) | 1 (Ref.)  0.67 (0.50-0.90)  0.97 (0.77-1.22)  0.86 (0.75-0.99) |
| Glucosamine  Non users  Current  Recent  Past | 15435 (98.56)  22 (0.14)  47 (0.30)  156 (1.00) | 79669 (98.50)  149 (0.18)  216 (0.27)  850 (1.05) | 1 (Ref.)  0.76 (0.49-1.19)  1.12 (0.82-1.54)  0.94 (0.79-1.12) | 1 (Ref.)  0.81 (0.51-1.28)  1.14 (0.82-1.57)  0.89 (0.74-1.06) |
| Chondroitin sulfate  Non users  Current  Recent  Past | 15433 (98.55)  32 (0.20)  57 (0.36)  138 (0.88) | 79500 (98.29)  277 (0.34)  313 (0.39)  794 (0.98) | 1 (Ref.)  0.59 (0.41-0.86)  0.94 (0.71-1.25)  0.90 (0.75-1.07) | 1 (Ref.)  0.61 (0.42-0.89)  0.94 (0.70-1.26)  0.81 (0.67-0.98) |

**Abbreviations:** CI: Confident Interval; OR: odds ratio.

**Definitions of different categories of CV risk:** High risk: patients with records of peripheral artery disease, angina pectoris, cerebrovascular accident or diabetes; intermediate risk: patients without criteria for high risk and with records of hypertension, dyslipidemia, chronic kidney failure, smoking or BMI>30kg/m^2^; low risk: the remainder.

*This stratified analysis was carried out using unconditional logistic regression, as the conditional logistic model showed high degree of instability.

^†^ Adjusted only for matching factors (age, sex, and calendar year).

^§^ Adjusted for: cerebrovascular disease (ischemic, hemorrhagic or unspecified stroke and transient ischemic attack), heart failure, angina pectoris (recorded as such, and/or use of nitrates), peripheral artery disease (PAD), hypertension, diabetes (recorded as such, and/or use of glucose-lowering medications), dyslipidemia (recorded as such, and/or use of lipid-lowering medications), rheumatoid arthritis, chronic kidney disease, asymptomatic hyperuricemia and gout, number of visits to the PCP in the year prior to the index date (as an indicator of comorbidities), body mass index (BMI), smoking, and current use of the following drugs: antiplatelet drugs, oral anticoagulants, nonsteroidal anti-inflammatory drugs (NSAIDs), paracetamol, metamizole, calcium with or without vitamin D supplements, corticosteroids, proton pump inhibitors (PPIs), H2-receptor antagonists, corticosteroids, angiotensin-converting enzyme inhibitors (ACEI), angiotensin II receptor blockers (ARB), calcium channel blockers (CCB), beta-blockers, alpha-blockers, and diuretics.

**S3 Table.** AMI risk associated with the use of SYSADOAs (Chondroitin Sulfate and Glucosamine) by history of angina pectoris.

| History of angina pectoris* | | | | |
| --- | --- | --- | --- | --- |
|  | **Cases (%)**  **N=2657** | **Controls (%)**  **N=5106** | **Non-adjusted**^†^ **OR (95%CI)** | **Adjusted OR**^§^ **(95%CI)** |
| SYSADOA (all)  Non users  Current  Recent  Past | 2557 (96.24)  7 (0.26)  29 (1.09)  64 (2.41) | 4840 (94.79)  45 (0.88)  65 (1.27)  156 (3.06) | 1 (Ref.)  0.27 (0.12-0.60)  0.79 (0.51-1.24)  0.73 (0.54-0.98) | 1 (Ref.)  0.29 (0.13-0.65)  0.92 (0.58-1.45)  0.83 (0.61-1.14) |
| Glucosamine  Non users  Current  Recent  Past | 2595 (97.67)  6 (0.23)  15 (0.56)  41 (1.54) | 4955 (97.04)  19 (0.37)  34 (0.67)  98 (1.92) | 1 (Ref.)  0.54 (0.22-1.37)  0.80 (0.43-1.47)  0.77 (0.53-1.12) | 1 (Ref.)  0.60 (0.23-1.46)  0.85 (0.45-1.61)  0.90 (0.61-1.33) |
| Chondroitin sulfate  Non users  Current  Recent  Past | 2604 (98.01)  1 (0.04)  17 (0.64)  35 (1.32) | 4960 (97.14)  27 (0.53)  17 (0.64)  83 (1.63) | 1 (Ref.)  0.07 (0.01-0.48)  0.83 (0.46-1.49)  0.74 (0.50-1.10) | 1 (Ref.)  0.06 (0.01-0.48)  1.04 (0.57-1.89)  0.83 (0.55-1.26) |
|  | | | | |
| No history of angina pectoris* | | | | |
|  | **Cases (%)**  **N=20928** | **Controls (%)**  **N=112299** | **Non-adjusted**^†^ **OR (95% CI)** | **Adjusted OR**^§^ **(95% CI)** |
| SYSADOA (all)  Non users  Current  Recent  Past | 20049 (95.80)  154 (0.74)  246 (1.18)  479 (2.29) | 107362 (95.60)  1116 (0.99)  1250 (1.11)  2571 (2.29) | 1 (Ref.)  0.74 (0.63-0.88)  1.06 (0.92-1.21)  1.01 (0.91-1.11) | 1 (Ref.)  0.71 (0.60-0.85)  0.98 (0.85-1.13)  0.92 (0.83-1.01) |
| Glucosamine  Non users  Current  Recent  Past | 20440 (97.67)  73 (0.35)  114 (0.54)  301 (1.44) | 109629 (97.62)  463 (0.41)  582 (0.52)  1625 (1.45) | 1 (Ref.)  0.85 (0.66-1.09)  1.06 (0.86-1.29)  1.01 (0.89-1.14) | 1 (Ref.)  0.85 (0.66-1.09)  1.00 (0.82-1.23)  0.92 (0.81-1.05) |
| Chondroitin sulfate  Non users  Current  Recent  Past | 20423 (97.59)  88 (0.42)  155 (0.74)  262 (1.25) | 109363 (97.39)  730 (0.65)  781 (0.70)  1425 (1.27) | 1 (Ref.)  0.65 (0.52-0.81)  1.06 (0.89-1.26)  0.99 (0.86-1.13) | 1 (Ref.)  0.61 (0.49-0.76)  0.98 (0.82-1.18)  0.89 (0.78-1.02) |

**Abbreviations:** CI: Confident Interval; OR: odds ratio.

**Definitions of different categories of CV risk:** High risk: patients with records of peripheral artery disease, angina pectoris, cerebrovascular accident or diabetes; intermediate risk: patients without criteria for high risk and with records of hypertension, dyslipidemia, chronic kidney failure, smoking or BMI>30kg/m^2^; low risk: the remainder.

*This stratified analysis was carried out using unconditional logistic regression, as the conditional logistic model showed high degree of instability.

^†^ Adjusted only for matching factors (age, sex, and calendar year).

^§^ Adjusted for: cerebrovascular disease (ischemic, hemorrhagic or unspecified stroke and transient ischemic attack), heart failure, angina pectoris (recorded as such, and/or use of nitrates), peripheral artery disease (PAD), hypertension, diabetes (recorded as such, and/or use of glucose-lowering medications), dyslipidemia (recorded as such, and/or use of lipid-lowering medications), rheumatoid arthritis, chronic kidney disease, asymptomatic hyperuricemia and gout, number of visits to the PCP in the year prior to the index date (as an indicator of comorbidities), body mass index (BMI), smoking, and current use of the following drugs: antiplatelet drugs, oral anticoagulants, nonsteroidal anti-inflammatory drugs (NSAIDs), paracetamol, metamizole, calcium with or without vitamin D supplements, corticosteroids, proton pump inhibitors (PPIs), H2-receptor antagonists, corticosteroids, angiotensin-converting enzyme inhibitors (ACEI), angiotensin II receptor blockers (ARB), calcium channel blockers (CCB), beta-blockers, alpha-blockers, and diuretics.

**S4 Table:** Risk of AMI associated with the use of SYSADOA (prevalent users included).

|  | Cases (%)  N=24155 | Controls (%)  N=120775 | Non-adjusted OR^†^  (95% CI) | Adjusted OR^§^  (95% CI) |
| --- | --- | --- | --- | --- |
| SYSADOA (all)  Non users  Current  Recent  Past | 22606 (93.59)  257 (1.06)  405 (1.68)  887 (3.67) | 112202 (92.90)  1882 (1.56)  2023 (1.68)  4668 (3.87) | 1 (Ref.)  0.68 (0.59-0.77)  0.99 (0.90-1.10)  0.94 (0.87-1.01) | 1 (Ref.)  0.68 (0.60-0.78)  0.96 (0.85-1.07)  0.89 (0.82-0.96) |
| Glucosamine  Non users  Current  Recent  Past | 23217 (96.12)  125 (0.52)  208 (0.86)  605 (2.50) | 115614 (95.73)  849 (0.70)  1037 (0.86)  3275 (2.71) | 1 (Ref.)  0.73 (0.61-0.88)  1.00 (0.86-1.16)  0.92 (0.84-1.00) | 1 (Ref.)  0.75 (0.62-0.91)  0.99 (0.85-1.15)  0.88 (0.80-0.96) |
| Chondroitin sulfate  Non users  Current  Recent  Past | 23322 (96.55)  145 (0.60)  235 (0.97)  453 (1.88) | 116140 (96.16)  1146 (0.95)  1174 (0.97)  2315 (1.92) | 1 (Ref.)  0.63 (0.53-0.75)  0.99 (0.86-1.15)  0.97 (0.88-1.08) | 1 (Ref.)  0.64 (0.53-0.76)  0.96 (0.83-1.11)  0.91 (0.82-1.02) |
| Chondroitin sulfate + Glucosamine*  Non users  Current  Recent  Past | 23933 (99.08)  13 (0.05)  80 (0.33)  129 (0.53) | 119552 (98.99)  110 (0.09)  361 (0.30)  752 (0.62) | 1 (Ref.)  0.59 (0.33-1.05)  1.11 (0.87-1.41)  0.86 (0.71-1.03) | 1 (Ref.)  0.63 (0.35-1.14)  1.09 (0.84-1.40)  0.83 (0.68-1.00) |

Abbreviations: OR: Odds ratio, CI: Confident Interval.

*Fixed-dose combination or concomitant use as separate drugs

^†^ Adjusted only for matching factors (age, sex, and calendar year).

^§^ Adjusted for: cerebrovascular disease (ischemic, hemorrhagic or unspecified stroke and transient ischemic attack), heart failure, angina pectoris (recorded as such, and/or use of nitrates), peripheral artery disease (PAD), hypertension, diabetes (recorded as such, and/or use of glucose-lowering medications), dyslipidemia (recorded as such, and/or use of lipid-lowering medications), rheumatoid arthritis, chronic kidney disease, asymptomatic hyperuricemia and gout, number of visits to the PCP in the year prior to the index date (as an indicator of comorbidities), body mass index (BMI), smoking, and current use of the following drugs: antiplatelet drugs, oral anticoagulants, nonsteroidal anti-inflammatory drugs (NSAIDs), paracetamol, metamizole, calcium with or without vitamin D supplements, corticosteroids, proton pump inhibitors (PPIs), H2-receptor antagonists, corticosteroids, angiotensin-converting enzyme inhibitors (ACEI), angiotensin II receptor blockers (ARB), calcium channel blockers (CCB), beta-blockers, alpha-blockers, and diuretics.

**S5 Table:** Evaluation of collinearity among the variables included in the adjusted model.

| Variable | VIF |
| --- | --- |
| SYSADOA | 1.05 |
| Visits to the PCPs | 1.43 |
| BMI | 1.32 |
| Smoking | 1.26 |
| CVA | 1.06 |
| Heart failure | 1.10 |
| Angina pectoris ^*^ | 1.13 |
| PAD | 1.04 |
| Hypertension | 1.64 |
| Diabetes ^†^ | 1.14 |
| Dyslipidemia ^§^ | 1.17 |
| Rheumatoid arthritis | 1.01 |
| Chronic kidney failure | 1.06 |
| Hyperuricaemia (asymptomatic) or gout | 1.12 |
| Antiplatelet drugs | 1.25 |
| Oral anticoagulants | 1.11 |
| Paracetamol | 1.17 |
| Metamizole | 1.16 |
| NSAIDs | 1.23 |
| Calcium suppl (w/, w/o vit D) | 1.07 |
| Corticosteroids | 1.09 |
| ACE inhibitors | 1.35 |
| ARB | 1.26 |
| CCB | 1.25 |
| Beta-Blockers | 1.15 |
| Alfa-Blockers | 1.06 |
| Diuretics | 1.25 |
| PPI | 1.22 |
| H_2_ receptor blockers | 1.03 |
|  | **Mean VIF = 1.18** |

Abbreviations: ACE: Angiotensin Converting Enzyme; ARB: Angiotensin II-Receptor Blockers; BMI: Body Max Index; CCB: Calcium-channel blockers; CVA: Cerebrovascular Accident; NSAIDs: Non-steroidal Anti-inflammatory Drugs; PAD: Peripheral Artery Disease; PCP: primary care physician; PPI: Proton-pump inhibitors; VIF: variance inflation factor.

^*^ Recorded as such or when patients were using nitrates.

^†^ Recorded as such or when patients were using glucose-lowering drugs.

^§^ Recorded as such or when patients were using lipid-lowering drugs.

**S6 Table.** Pattern of comorbidities, risk factors and baseline comedications among users of chondroitin sulfate or glucosamine and non-users of SYSADOAs.

|  | Current users of CS (%) | Current users of glucosamine (%) | Non-users of SYSADOAs (%) |
| --- | --- | --- | --- |
| Males | 58.26 | 54.36 | 72.54 |
| Hypertension | 52.58 | 51.66 | 42.66 |
| 70+ years | 44.52 | 48.96 | 48.69 |
| Dyslipidemia | 44.12 | 40.46 | 34.58 |
| PPIs use | 41.48 | 42.53 | 19.97 |
| Osteoarthritis | 28.80 | 31.12 | 6.96 |
| NSAIDs use | 25.76 | 29.25 | 8.46 |
| Paracetamol use | 22.19 | 28.42 | 11.49 |
| BMI 30+ | 29.99 | 29.25 | 19.30 |
| ARBs Use | 21.40 | 15.98 | 11.71 |
| Asymptomatic hyperuricemia | 19.15 | 19.80 | 14.56 |
| ACEIs use | 18.89 | 17.22 | 14.19 |
| Diabetes | 17.31 | 16.60 | 16.44 |
| Smoking | 15.06 | 12.03 | 16.94 |
| Antiplatelets use | 14.00 | 13.07 | 12.04 |
| CCBs use | 12.95 | 13.49 | 9.33 |
| Diuretics use | 11.10 | 12.24 | 10.29 |
| Calcium supplements use | 11.49 | 10.17 | 3.18 |
| Beta blockers use | 7.27 | 6.22 | 6.24 |
| CVA | 5.94 | 4.15 | 5.30 |
| Metamizole use | 5.42 | 4.56 | 2.72 |
| Gout | 5.02 | 3.32 | 4.27 |
| Anticoagulants use | 4.10 | 3.32 | 4.15 |
| Angina pectoris | 3.57 | 3.94 | 4.31 |
| Alpha blockers use | 2.91 | 2.49 | 2.03 |
| Chronic renal failure | 2.91 | 2.07 | 2.41 |
| Heart failure | 1.85 | 1.24 | 2.62 |
| Corticosteroids use | 1.59 | 1.66 | 1.44 |
| H2-Receptor antagonists use | 1.06 | 2.28 | 1.36 |
| PAD | 1.45 | 0.21 | 2.08 |

Abbreviations: ACE: Angiotensin Converting Enzyme; ARB: Angiotensin II-Receptor Blockers; BMI: Body Max Index; CCB: Calcium-channel blockers; CS: Chondroitin Sulfate; CVA: Cerebrovascular Accident; NSAIDs: Non-steroidal Anti-inflammatory Drugs; PAD: Peripheral Artery Disease; PPI: Proton-pump inhibitors.

**S1 Figure.** Pattern of comorbidities, risk factors and baseline comedications among users of chondroitin sulfate or glucosamine and non-users of SYSADOAs.


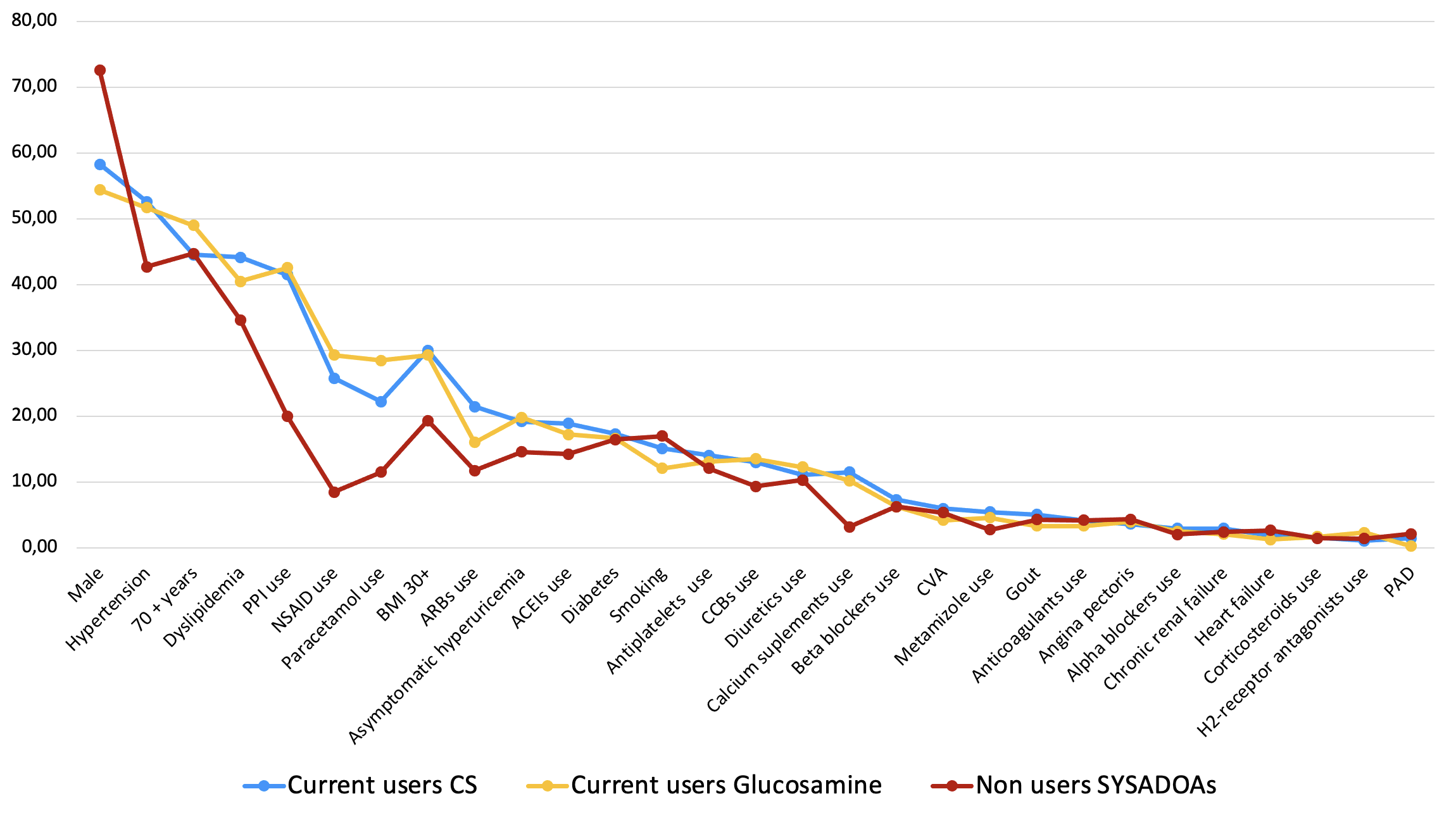


Abbreviations: ACE: Angiotensin Converting Enzyme; ARB: Angiotensin II-Receptor Blockers; BMI: Body Max Index; CCB: Calcium-channel blockers; CS: Chondroitin Sulfate; CVA: Cerebrovascular Accident; NSAIDs: Non-steroidal Anti-inflammatory Drugs; PAD: Peripheral Artery Disease; PPI: Proton-pump inhibitors.
